# Why are babies born before arrival at health facilities in King Sabata Dalindyebo Local Municipality, Eastern Cape, South Africa? A qualitative study

**DOI:** 10.4102/phcfm.v7i1.881

**Published:** 2015-11-09

**Authors:** Adeyinka A. Alabi, Don O’Mahony, Graham Wright, Mohlomi J. Ntsaba

**Affiliations:** 1 Department of Family Medicine, Faculty of Health Sciences, Walter Sisulu University, South Africa; 2 School of Health Sciences, University of Fort Hare, East London, South Africa; 3 Department of Nursing, Faculty of Health Sciences, Walter Sisulu University, South Africa

## Abstract

**Introduction:**

Babies born before arrival at a health facility have a higher risk of neonatal death and their mothers a higher risk of maternal death compared with those born in-facility. The study explored the reasons for mothers giving birth before arrival (BBA) at health facilities and their experiences of BBA.

**Methods:**

A qualitative research design was used. Individual and focus group interviews of BBA mothers and of nurses were undertaken at a community health centre and a district hospital in King Sabata Dalindyebo Local Municipality.

**Results:**

Reasons for BBA included a lack of transport, a lack of security at night that deterred mothers from travelling, precipitate labour, failure to identify true labour, and a lack of waiting areas at health facilities. Traditional and cultural beliefs favouring childbirth at home and nurses’ negative attitudes during antenatal care and labour influenced mothers to go to health facilities when in advanced labour. Mothers were aware of possible complications associated with BBA.

**Conclusion:**

Socio-economic, individual, cultural and health system factors influence the occurrence of BBA. Relevant parties need to address these factors to ensure that all babies in the King Sabata Dalindyebo Local Municipality are delivered within designated health facilities.

## Abstrait

**Pourquoi les bébés naissent-ils avant d’arriver aux centres médicaux de la municipalité locale de King Sabata Dalindyebo, dans l’Eastern Cape, en Afrique du Sud ? Etude qualitative.**

**Introduction:**

les bébés qui naissent avant d’arriver au centre médical ont un risque plus élevé de mort néonatale et leur mère un risque plus élevé de décès maternel que ceux qui sont nés dans l’établissement. L’étude a examiné pour quelles raisons les mères accouchent avant d’arriver à la clinique (AAA) et leur expérience d’AAA.

**Méthodes:**

On a utilisé un modèle de recherche qualitative. On a mené des entrevues individuelles et de groupes témoins des mères AAA et des infirmières à un centre médical communautaire et à un hôpital de district de la municipalité de King Sabata Dalindyebo.

**Résultats:**

Les raisons d’AAA étaient le manque de transport, l’insécurité la nuit qui empêchait les mères de se déplacer, l’accouchement subi, l’incapacité de reconnaitre l’accouchement véritable, and le manque de salles d’attente dans les centres médicaux. Les croyances traditionnelles t culturelles en faveur de l’accouchement à la maison et l’attitude négative des infirmières envers les soins prénatals et l’accouchement ont influencé les mères d’aller au centre médical quand elles sont prêtes à accoucher. Les mères savaient qu’il pouvait y avoir des complications dues à l’AAA.

**Conclusions:**

des facteurs socio-économiques, individuels, culturels et du système de santé influence l’incidence de l’AAA. Les acteurs appropriés doivent remédier à ces facteurs pour faire en sorte que tous les bébés de la municipalité de King Sabata Dalindyebo naissent dans les établissements désignés de soins de santé.

## Introduction

Neonatal mortality rates are highest in developing countries (22 per 1000) compared with those in developed countries (3 per 1000),^1^ with the major causes of death being prematurity and birth asphyxia.^2,3^ The neonatal mortality rate in South Africa is high at 15 per 1000 live births, and neonatal deaths constitute 40% of under-5 deaths, with most deaths occurring in the early neonatal period.^4^ South Africa is committed to the United Nations’ Millennium Development Goals (MDGs) to reduce by two-thirds the under-5 mortality rate and by three-quarters the maternal mortality ratio between 1990 and 2015. The under-5 mortality rate in 2013 was 45 per 1000 live births compared with the MDG target of 20.^5^ Stillbirth rates are also highest in non-developed regions (29 per 1000 births) compared with high-income countries (3 per 1000).^6^ In South Africa, the rate in 2013 was 17 per 1000.^7^ Similarly, maternal mortality is high in developing countries (233 per 100 000) compared with developed countries (12 per 100 000),^8^ with the major causes of death being hypertension, haemorrhage and sepsis.^9^ In South Africa, the maternal mortality ratio was 140 per 100 000 in 2013, whilst the MDG-5 target was 38,^5^ and HIV is an additional, major cause of maternal death.^10^ Whilst South Africa is unlikely to attain the MDG targets, every effort should be made to do so. To reduce stillbirths and neonatal and maternal mortality, all births should take place in health facilities with skilled birth attendants and means of transfer for emergencies.^11,12^

Birth before arrival (BBA), defined as delivery of a baby that takes place outside healthcare facilities,^13^ is associated with increased neonatal mortality and morbidity, both in developed^14,15^ and developing countries.^13,16^ The BBA rate in developed countries is less than 1%,^17,18^ but the rate in South Africa is 10%.^19^ At Mthatha General Hospital and Ngangelizwe Community Health Centre (CHC), both situated in King Sabata Dalindyebo Local Municipality, the rate was 4% and 7%, respectively, in 2012 (P Yogeswaran, personal communication, 30 January 2013). These rates are higher than 1.5%, a rate considered acceptable in South Africa by Potter et al.^20^ Previous studies in South Africa have described factors influencing delivery in health facilities.^21,22,23^ However, the authors found only one study that included qualitative interviews with BBA mothers in South Africa.^23^ The present study explored perceptions of mothers and nurses about the contributory factors to BBA so as to inform interventions to reduce the BBA rate in King Sabata Dalindyebo Local Municipality.

## Research methods and design

A qualitative approach was chosen as it would ‘enable participants to speak for themselves’^24^ and provide a deeper understanding of factors associated with BBA.

### Setting

Both Mthatha General Hospital and Ngangelizwe CHC, with an average of 500 and 200 deliveries per month respectively, are located in the town of Mthatha, situated in the predominantly rural Oliver Reginald Tambo District, the third most deprived of 52 districts in South Africa.^25^ Indicators of deprivation include the following: an estimated 59% of the population live below the poverty line; the unemployment rate is high at 44%; there is no access to piped water for 51% of households; 70% live in traditional dwellings; and 30% of households have no electricity.^26^

### Sampling

Purposeful sampling was used to select BBA mothers and nurses for interview. The most senior nurse in charge selected participants after assessing that they were likely to freely and fluently express their views. BBA mothers who presented within 3 months of delivery were recruited. The sample size was determined by data saturation, namely, when no new themes or insights were obtained, recruitment was stopped.^24^ The inclusion criterion for the nurses was that they must have worked at one of the study facilities for at least 1 year and must have attended to BBA mothers.

### Data collection methods

For the mothers, individual in-depth interviews were conducted, whilst two focus group interviews were undertaken with the nurses, one at each site. A female research assistant trained in qualitative interviewing conducted semi-structured interviews with BBA mothers in their first language (isiXhosa), either at the hospital or at the CHC. An interview guide, based on themes identified in the literature review, was used. The guide was piloted by interviewing two mothers and minor clarifications were made to the guide. The two interviews were not included in the study. Open-ended questions were used to allow participants to freely express their views. However, probing and prompting of participants were undertaken, where necessary, to obtain clarity of information.

One author (A.A.A.) facilitated the focus group interviews, whilst the research assistant took field notes. Focus groups use group dynamics and different forms of communication such as teasing, jokes and arguing to explore views.^27^ The researchers considered that focus groups would be sufficient for clarifying nurses’ views. The focus groups were conducted in English. All the interviews were audiotaped and field notes were taken by the interviewer to record any emotions displayed or behavioural cues.

### Data analysis

The research assistant transcribed audiotaped recordings of the interviews and field notes and translated the individual interviews into English. To ensure that transcription was accurate, an educator whose first language was isiXhosa cross-checked excerpts of the transcriptions with the recordings.

The ‘framework approach’ was used to inductively analyse data.^28^ A ‘cut and paste’ using a word processor was used to group selection of data. Themes emerging from the focus group discussions and the in-depth interviews were combined, compared and triangulated using content analysis.

### Trustworthiness

Four constructs were utilised to ensure the trustworthiness of the present study.^29^ For credibility, triangulating of data collection methods was utilised (focus group and in-depth interviews) as well as triangulation of sources of the data by interviewing BBA mothers and nurses. The validity of data collected was ensured by having an independent translator randomly verify the accuracy and completeness of the excerpts from the transcripts with the audiotaped interviews and the translation of transcripts. For transferability, sufficient, rich and thick descriptions are given for readers to understand if the results can apply to other settings. For dependability, established research methodology was used with sufficient detail to allow a reader to judge its reliability. For confirmability, triangulation was used to reduce the effect of possible researchers’ bias. The author A.A.A. is a medical practitioner trained in a predominantly biomedical paradigm, whilst the research assistant (Phelo Sithole) is a graduate in social sciences.

## Ethical considerations

Ethical clearance was obtained from Walter Sisulu University Health Research Ethics and Bio-Safety Committee (Protocol No. 014/2013) and permission to carry out the study was obtained from the Department of Health, Eastern Cape Province. Informed consent was obtained from all participants. It was emphasised that they could stop participation at any time without giving a reason and without prejudicing their relationship with their healthcare providers. They were reassured that their participation was confidential as all interviews were available to researchers only and that numbers, instead of names, were to be used for analysis and publication. For one 16-year-old participant, consent was obtained from her and her grandmother.

## Results

Nineteen mothers were interviewed. Six nurses participated in the CHC focus group and 9 in the hospital group.

**Socio-demographic characteristics of mothers and details of BBAs:** These are displayed in Table 1. The median age of mothers was 27 years with a range of 16–41 years. One had completed grade 12, 15 were single and 18 were unemployed at the time of the study. Seventeen were multiparous. One mother had all her deliveries as BBAs.

**TABLE 1:** Socio-demographic characteristics of mothers and details of birth before arrival.

Participant	Age (years)	School level^†^	Marital status	Employed (Y/N)^‡^	Parity	Previous BBA (Y/N)	Travel time to facility (minutes)^§^	Time of birth
1	28	9	Married	N	3	N	50	03h00
2	22	11	Single	N	4	N	30	01h00
3	18	8	Single	N	1	N	30	04h00
4	25	4	Single	N	4	N	about 60	23h00
5	35	11	Single	N	4	N	90	15h00
6	19	5	Single	N	2	Y	30	22h00
7	20	11	Single	N	2	N	30	13h00
8	33	10	Married	Y^¶^	6	Y	30	10h00
9	28	3	Cohabiting	N	2	N	120	03h00
10	16	11	Single	N	1	N	20	05h00
11	41	11	Single	N	6	N	60	01h00
12	32	7	Married	N	4	N	30	01h00
13	21	9	Single	N	2	N	45	03h00
14	27	6	Married	N	3	Y	30	04h00
15	23	11	Single	N	2	N	60	06h00
16	34	9	Single	N	5	Y	30	12h20
17	29	10	Single	N	3	N	50	22h00
18	25	8	Single	N	4	N	90	02h00
19	30	3	Single	N	5	N	30	17h30

BBA, birth before arrival.

†, Highest school grade (range 1–12); ‡, Y = yes; N = no; §, A health facility designated to conduct births; ¶, Employed as a domestic worker.

**Details of delivery:** Fourteen of the participants delivered at home, assisted by family or community members. Two deliveries occurred in an ambulance and one in a private car. One woman delivered in an open public space whilst walking to the clinic, whilst another delivered just outside the Mthatha General Hospital's Outpatient Department.

**Birth outcomes:** There were two early neonatal deaths. The mothers reported that the neonates died within minutes of birth.

**Planned place of birth:** Seventeen of the 19 mothers planned to deliver in either a community health centre (CHC) or hospital, whilst the remaining two wanted to deliver at home.

## Qualitative findings

Five themes were identified, the first four of which were risk categories for BBA. Theme five relates to mothers’ understanding of risks associated with BBA. Quotes from the participants were assigned codes as follows: each participant was assigned a number. C and H codes represent Ngangelizwe CHC and Mthatha General Hospital, respectively. For example, in the first quotation below, the number 5 indicates participant number 5, H indicates the site (Mthatha General Hospital) and the interviewee is a mother who is 27 years old.

## Theme 1: Physical barriers to accessing a health facility

Accessibility to health facilities was affected by distance, bad roads, and transport and financial constraints.

### Distance

About using a taxi or hired van to reach the health facility from home, one mother reported that it took her less than 30 minutes, whilst the remaining 18 reported that it took 30 or more minutes:

‘The nearest clinic to us does not open at night. So the only option available for us was to go to Mthatha General, which would take a commercial vehicle more than one hour to reach. We called the emergency ambulance but I had already delivered by the time they came at 1 a.m.’ (PH5, mother, 27)

Nurses also felt that most mothers came from locations that require transport to reach the health facility.

### Poor roads

Another major issue identified was the bad state of the untarred roads, making access difficult, especially when it rained:

‘The road to some of the locations where the mothers are staying are in a very terrible situation most especially if it is raining, this makes it very difficult for ambulances to reach those locations, even when they received emergency call from a mother in labour.’ (PC2, nurse, 42)

### Transport

Mothers and nurses blamed the poor response time by the public Emergency Medical Services (EMS) ambulance service for BBA. They estimated that the average response time was between 2 and 3 hours. Factors perceived as contributing to the delay were a shortage of ambulances and drivers, and drivers having difficulty in locating respondents’ houses in rural areas. Nine mothers called an ambulance when in labour. One mother's experience was as follows:

‘My mother called the ambulance at about 3 a.m. The driver could not get the description and he was lost for about two hours. By the time he arrived in our place it was 6 a.m. and I had already delivered.’ (PH4, mother, 21)

Another reported that the ambulance personnel were impatient:

‘The ambulance came some minutes after we called. We saw the ambulance parking adjacent to our house and just before we crossed to where it was, it zoomed off. I ended up delivering with the assistance of old ladies at home.’ (PH1, mother, 16)

Ten mothers could not contact the EMS when they were in labour: six did not know the phone number, two did not have airtime, and two had flat batteries in their mobile phones.

Nurses identified poor EMS management and a lack of resources:

‘We sometimes got the response from the EMS call centre that the ambulances have been taken for repair or there is no driver or the licence disks have not been renewed, so they are not working …’ (PC1, nurse, 51)

Because of delays by the EMS, one mother reported that she was advised at the clinic to save money so that she can hire a vehicle in case of emergency:

‘They told us that we should have an arrangement for a car that can take us to the hospital when we fall in labour. The nurses told us to have money saved for transportation in case of emergency.’ (PH6, mother, 23)

Only one mother was transported by a neighbour. In the absence of an ambulance, mothers stated that neighbours were usually of no help in transporting them to a health facility:

‘The rate of crime and hijacking is the problem … that is why people with cars refuse to take people to the hospital, especially at night.’ (PH8, mother, 29)

Three mothers reported the challenges of getting a taxi at night:

‘I did not see any taxi to take me to the hospital because it was already late and our public transport was about to stop working for the day.’ (PC7, mother, 20)

### Financial constraints

Nurses felt that most mothers were poor. At the time of the study, 95% were unemployed (Table 1). Four of the mothers also attributed a lack of money to hire a taxi at night as being responsible for their home birth:

‘I delivered at home because the ambulance did not come and a cab driver charged me R400 which I did not have.’ (PC8, mother, 33)

## Theme 2: Traditional delivery beliefs and practices

**Traditional medicines:** Nurses perceived that many mothers used traditional medicines to speed up labour. One of the nurses reported that she herself had used traditional medication to hasten childbirth in the past. Nurses thought that BBA mothers were usually not willing to disclose such information until complications arose during labour:

‘They only disclose use of traditional medicine when they are in trouble during delivery.’ (PC5, nurse, 50)

However, only one mother agreed that she used traditional medicines:

‘I took one bottle of traditional medicine ‘umchamo wemfene’ [*baboon's urine*] at nine months because there were no signs that I was going to deliver anytime soon and the elders believed that I was being bewitched.’ (PC5, mother, 35)

**Place of childbirth:** Nurses perceived that Xhosa traditions and beliefs contributed to BBA:

‘There is a belief in our tradition that if you deliver in the health facility you are not the real mother; a real mother has to deliver at home.’ (PH6, nurse, 40)

Tradition also determines who takes the final decision on the place of delivery. The wish of the parturient does not count:

‘A parturient may be educated but because of the tradition you have to give birth at home. You are not going to tell the elders on what to do.’ (PH3, nurse, 56)

**Traditional childbirth:** Mothers reported that family members and neighbours, usually elderly women, assisted them during delivery. One mother described her labour and delivery:

‘My grandmother gave me an empty bottle and told me to blow into it. She said this helped the baby to be active. She also used hot water to massage the tummy with the hope that this assists the baby to go down. [*During delivery*] I was on my knees and I only know now that it is not supposed to be happening like that. The right position is to lie with my back.’ (PC3, mother, 18)

Nurses reported that parturients assume a squatting position during home delivery unlike in hospital where parturients lie on their backs:

‘Some of these women are not used to hospital method of giving birth where they are requested to lie on their back, they are used to squatting, so in that case they do not like going to the hospital.’ (PH1, nurse, 55)

If BBA mothers come with retained placentae, nurses discern the traditional method of delivering the placenta by tying a bottle to the umbilical cord:

‘They usually do not tell us that a traditional birth attendant did the delivery but we can tell because we see a bottle tied up in the thigh with the placenta, in the traditional method of removing the placenta.’ (PC6, nurse, 28)

**Preference for female birth attendant:** Four nurses reported that parturients often expressed a preference for female birth attendants:

‘They equally do not like to be helped by a male nurse or male doctor.’ (PH4, nurse, 50)

## Theme 3: Health system-related issues

### Antenatal care

**Poor antenatal attendance:** The majority (18) of mothers reported that they attended the antenatal clinic at least once during their pregnancy. However, 15 booked late (after 20 weeks) and 14 did not complete their antenatal clinic schedule. One mother felt that there was no benefit from attending antenatal care. Two gave reasons for poor attendance:

‘It was difficult for me to attend clinic regularly because I was working as a helper and my boss would sack me if I were to be taken permission for every visit.’ (PC8, mother, 33)

‘I started attending antenatal clinic at 6 months but I could not attend regularly because I was writing examinations.’ (PC3, mother, 18)

Two nurses reported that teenagers are afraid to report pregnancy for fear of being reprimanded by their parents:

‘Some of the teenagers usually open up to their parent at a very late stage of labour leading to giving birth en-route to the hospital.’ (PC3, nurse, 42)

**Quality of health education:** Ten mothers reported that nurses educated them about signs and symptoms of labour:

‘The nurses told us to prepare ourselves when we are about to give birth by sorting and making ready the baby's clothes. They equally warned us not to depend on the ambulance because it is mostly used to transport sick people.’ (PC2, mother, 22)

However, only 6 mothers understood the components of birth preparedness taught during antenatal care, namely when, how and where to go in labour.

**Lack of resources at clinics:** Two mothers saw no benefit in attending an antenatal clinic as antenatal cards were out of stock:

‘The problem with the clinic is that they are always out of clinic cards. That is why I did not visit regularly.’ (PH3, mother, 21)

### Poor attitudes of nurses

Two mothers blamed the poor attitudes of nurses at the health facility for having a BBA. One mother was influenced by the perceptions of a relative:

‘I only went to the clinic at 6 months and I did not go back again. I was afraid because my sister scared me that nurses usually assault women that started booking late.’ (PC6, mother, 19)

Another mother waited until an advanced stage of labour before going to the health centre as she had personal experience of poor attitudes:

‘I was so scared of going to the clinic when I was in early labour because I have no clinic card. Nurses are usually very angry and shout at us if we do not have a card and I did not want to be shouted at. So I decided to wait till close to the delivery time before going, but I ended up giving birth at home.’ (PH2, mother, 41)

### Poor quality of care

Four mothers blamed nurses for sending them back home when they presented with labour pains:

‘After the initial examination by the nurse, she told me that I was not in labour and that I should go back home. She should have allowed me stay and observe me until she was sure about what was going on with me; rather, she sent me back home only for me to delivered at midnight at home when there was no transport.’ (PC9, mother, 28)

One mother became visibly upset whilst narrating her ordeal and a counselling session was organised for her after the interview:

‘I went to the clinic which I paid 10 Rands taxi fare, where I used to go for ANC to take my ARV/nevirapine. When I got there the nurses were in a meeting that took 3 hours. When the nurses came out of their meeting I was told that the nevirapine was out of stock and I should try another clinic which was a bit far from home, which I had to pay an extra 15 Rands to get to. It was already late in the afternoon and because of the high rate of crime I could not go back home and had to ask for a place to sleep near the clinic. I went to bed and unfortunately for me the labour pains started at 9 p.m., I could not reach the EMS because my phone battery was flat and there was no taxi to take me to the clinic. I delivered at 3 a.m. … The baby stopped moving after about 30 minutes of delivery.’ (PC1, mother, 28)

Five CHC nurses felt that a shortage of beds (for observation) contributes to BBA:

‘We discharge them when we see they are not in true labour since we do not have enough beds.’ (PH5, nurse, 42)

The nurses also felt that a shortage of experienced staff at night could lead to a wrong diagnosis of false labour:

‘Another problem is the low level and poor quality of experience of the nurses. I am saying this because you will find that the patient is examined by an inexperienced nurse who is supposed to be working under supervision … and she misdiagnoses a true labour as a false labour and subsequently sends the patient home.’ (PC9, nurse, 50)

## Theme 4: Unanticipated events resulting in birth before arrival

### Unsure of labour

Three mothers attributed their BBA to being unsure of when they were in labour:

‘I started feeling abdominal pain during the day while doing laundry; however, the pain was not serious and it was on and off. My water broke around 8 p.m. and I was going to pee frequently. My grandmother suspected and as soon as she asked me to kneel down, the baby was already coming out.’ (PH9, mother, 25)

### Miscalculation of dates

One mother reported that being unsure of dates was the reason for BBA:

‘I guess I delivered at home because I thought I was in my eight month of pregnancy not knowing I was already at the 9 month.’ (PC4, mother, 25)

### Precipitate labour

Eight mothers reported that they delivered at home or en route to the hospital because the labour was too fast:

‘I started to feel the pains around 9 p.m. at night. I felt the pain at the lower back and I told those with me at the time. They went out to find the ambulance numbers, but by the time they came back my water broke and I had already delivered. The labour took less than an hour.’ (PH10, mother, 30)

### Preterm delivery

Three mothers explained that they had delivered before 8 months of pregnancy. One of these babies died and the other two were transferred to hospital. One described her experience as follows:

‘I gave birth to a live baby that was covered with sac membrane, but the strange thing was that she never cried despite moving the legs. The baby stopped moving after about 30 minutes of delivery. I think the membrane that covered the head and body of baby killed her. I was clueless about how to remove the sac.’ (PC1, mother, 28)

Another mother narrated her experienced as follows:

‘I was 7 months pregnant when I felt the pain during the day and I told my aunt. She said we should take a walk and by the time we came back I felt like sitting down. I did that and went for a pee and the blood came out followed by a lot of water then after that the baby came. … The nurses told me the baby was ill and has been admitted to Mandela [*Hospital*].’ (PH3, mother, 21)

## Theme 5: Risks associated with birth before arrival

The numbers of mothers who actually experienced complications were as follows: placenta was retained in eight, four of which were removed at a health facility; excessive blood loss was seen in seven, of whom one required a blood transfusion; perineal tears that required suturing at a health facility in five; and two neonatal deaths occurred.

Seventeen mothers perceived that a delivery outside a health facility could be dangerous. Four mothers identified that children may need suction of secretions from the mouth and nostrils:

‘The child might get sick, because things that are removed at the clinic will not be removed. Things like removing thick saliva in the mouth.’ (PC4, mother, 25)

Six mothers expressed a fear of injury with malpresentation or of the baby falling during home delivery:

‘The child might die due to little knowledge of traditional midwives, the child might be paralyzed and maybe the child might come with a wrong position or fall during delivery.’ (PH7, mother, 34)

Other risks identified were HIV transmission and the baby getting cold. One mother who experienced an early neonatal death described a complication consistent with hypothermia:

‘There was delay in the arrival of the EMS and I had delivered two hours before they eventually came. Because it was winter time, the weather was very cold and we wrapped the baby in the blanket. At hospital, the nurses told me that the child had caught too much cold and he did not make it.’ (PC9, mother, 28)

## Discussion

Most of the mothers interviewed had intended to deliver either in a CHC or in hospital. They had a good understanding of risks associated with BBA. Some of the participants actually experienced complications such as perineal tears, retained placenta, postpartum haemorrhage and neonatal death.

Mothers and nurses identified several factors that influenced the risk of BBA. The majority of BBA mothers reported that they stayed between 30 and 120 minutes’ drive from a health facility. These findings were similar to those in a mixed urban-rural setting in Nepal, where a distance of more than 1 hour to hospital was associated with an increased risk of home delivery.^30^ Similarly, Blondel et al.^31^ in France found that the risk of BBA was strongly associated with distance to the closest maternity unit. In contrast, Parag et al.^32^ did not find any association between distance from a health facility and BBA in a peri-urban setting in South Africa.

Transportation was identified as the major source of delay in reaching a health facility. Issues identified were a poor EMS ambulance service, bad roads, a lack of safety at night and an absence of taxis especially at night. Two mothers incorrectly thought they needed to pay for airtime to call the EMS, although emergency calls are free in South Africa. Challenges were encountered in engaging the services of commercial and private vehicle owners. The difficulty of obtaining transport at night is supported by the finding that 15 of the 19 mothers delivered between 22h00 and 06h00. This finding is consistent with results from other studies,^32,33^ where most BBAs occurred at night. In rural South Africa, a lack of transport contributed to BBA.^23^ Similar results have been reported from Nepal^34^ and Brazil.^35^

Whilst mothers were not asked directly about household income and affordability of transport, nurses reported that most BBA mothers were poor. The mothers’ demographic characteristics show that only one completed grade 12, and the majority were single and unemployed. The findings were similar to those of a study of 19 states in the USA, where BBA mothers were more likely to be single and uneducated.^18^ In contrast, Potter et al.^20^ did not find any significant difference in educational level and marital status between mothers who had BBA and mothers who delivered in hospital in a predominantly urban area of South Africa.

Mothers blamed nurses for sending them home when they presented with labour pains. However, nurses noted that a lack of facility beds prevented them from admitting women for observation. Nurses identified a shortage of experienced nurses, leading to poor supervision of junior nurses such that they misdiagnosed true labour as false labour. Studies in Brazil^35^ and Nepal^33^ also identified the failure of health personnel to identify a woman in early labour as contributory to BBA.

Mothers reported that unforeseen events contributed to BBA, such as miscalculation of dates, being unsure of labour, precipitate labour and preterm labour. Fast labour was associated with BBA in South Africa^20^ and Nepal,^33^ whilst preterm labour was associated with BBA in South Africa.^32^

Currently, there is no maternity waiting home at Mthatha. Maternity waiting homes can be used by pregnant women to await labour if they anticipate transport difficulties, if nurses are unsure they are in labour or if there are risk factors for preterm or precipitate labour. Developing such homes is an accepted part of the policy of the Department of Health in South Africa.^36^

One woman described using traditional medicines to induce labour. However, the nurses perceived that many mothers use traditional medicines. An example is ‘baboon's urine’ (isiXhosa: *umchamo wemfene*) that is used to ease labour.^37^ Nurses believe it can speed up labour, leading to BBA. In Africa, traditional medicines are commonly used to hasten labour and may cause adverse outcomes in pregnancy.^38,39^ Nurses felt that mothers do not usually volunteer such information to health workers for fear of being reprimanded. Judgemental attitudes by healthcare workers have been noted in studies on traditional medicines.^39^

Nurses reported that Xhosa traditions can preclude a parturient from utilising a health facility for childbirth, as a parturient who delivers in hospital is not considered a real mother. Similar findings were described in Uganda.^40^

Nurses stated that, in traditional home childbirth, a woman assumes a squatting position, unlike in hospital where parturients deliver in the supine position. They suggested that this may influence mothers to choose home delivery. However, this view did not emerge during the in-depth interviews, although three mothers mentioned that they used a squatting position during delivery. A study of home deliveries in the Eastern Cape, South Africa, found that 92% of women adopted an upright position during delivery.^41^ In the Lao People's Democratic Republic, one of the reasons why mothers chose home delivery was a preference for the traditional squatting position.^42^ A systematic review of use of the upright position in delivery suggested several possible benefits, including a reduction in assisted deliveries and episiotomies.^43^

Nurses reported that parturients expressed a preference for female birth attendants, which implies that women should be educated on the gender composition of the healthcare workforce and the midwifery skills that are common to both.

Mothers expressed concerns about nurses’ attitudes and behaviour such as shouting at parturients and a lack of prompt attention during antenatal care. This disrespectful care deters women from delivering in health facilities. Similar findings have been described in South Africa^23,44^ and Sub-Saharan Africa.^45,46,47,48^ Efforts are underway in South Africa^44^ and internationally to improve attitudes.^49^

A lack of necessary materials such as stationery and antiretroviral drugs also contributed to the perceived poor quality of services at the health facilities. This view is consistent with the findings of Mselle et al.,^46^ who found that inadequate resources contributed to low utilisation of health facilities in Tanzania.

## Limitations

As the present study was qualitative, the results cannot be generalised to the whole OR Tambo District Municipality; however, they may be transferable to similar settings.

Conducting interviews in the health facilities might have restricted mothers from freely discussing issues that related to failure of the medical services. Whilst interviews conducted at their homes would be optimal, it would have been expensive and time consuming to do so.

## Conclusion

The study identified that all but two BBA mothers intended to deliver in a health facility. Reasons identified as contributing to BBA were poor physical access to health facilities (transport and roads); negative attitudes by nurses; inadequate nursing skills in identifying true labour; culture and tradition favouring home delivery; inadequate recognition by mothers of labour; and preterm and precipitate labour. Mothers booked late and did not attend for the recommended number of antenatal care visits.

## Recommendations

Recommendations to the Department of Health to improve the health system for mothers and babies include the following points. A maternity waiting home is needed at Mthatha for women who may encounter difficulties in accessing a health facility during labour and for women who may be in early labour. Provision of a functional, dedicated obstetric ambulance system can reduce delays in transporting parturients to health facilities.^50^ A programme should be implemented to improve nurses’ attitudes, based on the universal rights of childbearing women^51^ and effective intervention programmes^44^ to improve nurses’ skills in diagnosing true labour and to allow parturients a choice of delivery position.^43^ Health education for pregnant women should focus on early booking, advice on cultural practices that may be detrimental to health, the signs and symptoms of labour, and when and how to go to an appropriate health facility for delivery.
